# Preoperative dynamic anterior tibial translation is not predictive of graft rupture after anterior cruciate ligament reconstruction

**DOI:** 10.1002/jeo2.70784

**Published:** 2026-05-31

**Authors:** David Mazy, Nicolas Cance, Lucia Angelelli, Tomas Pineda, Michael James Dan, David Henri Dejour

**Affiliations:** ^1^ Orthopedic Surgery Department, Lyon Ortho Clinic Clinique de la Sauvegarde Lyon France; ^2^ Clinica Ortopedica e Traumatologica 2 IRCCS Istituto Ortopedico Rizzoli Bologna Italy; ^3^ Hospital del Trabajador, Facultad de Medicina Universidad Andrés Bello Santiago Chile; ^4^ Hospital el Carmen, Facultad de Medicina Universidad Finis Terrae Santiago Chile; ^5^ East Coast Athletic Orthopaedics, Macquarie and Lingard Hospital Sydney New South Wales Australia

**Keywords:** anterior cruciate ligament, laximetry, meniscus, posterior tibial slope, static anterior tibial translation

## Abstract

**Purpose:**

This study aimed to determine whether higher preoperative dynamic anterior tibial translation (DATT) using laximetry is a risk factor for graft rupture after anterior cruciate ligament reconstruction (ACLR).

**Methods:**

This retrospective study included all patients who underwent primary ACLR with hamstring autograft between January 2014 and December 2017. Demographic data, absolute DATT on the injured side and ΔDATT (side‐to‐side difference), posterior tibial slope (PTS), static anterior tibial translation (SATT), concomitant lateral extra‐articular tenodesis (LET) and meniscal tears were collected. Subgroup analysis was performed using a ΔDATT threshold of 6 mm. Univariate and multivariable logistic regression analyses were conducted to identify independent risk factors for ACL graft rupture.

**Results:**

Among the 680 patients included with a minimum follow‐up of 6 years, 41 (6%) experienced graft rupture at a mean of 45 ± 30 months postoperatively. The median DATT on the injured side was 9 mm [interquartile range, IQR, 5], and the median ΔDATT was 6 mm [IQR, 4]. Graft rupture occurred in 5.8% for patients with ΔDATT < 6 mm and in 6.3% for patients with ΔDATT ≥ 6 mm (*p* = 0.787). Patients with ΔDATT ≥ 6 mm demonstrated a higher prevalence of medial meniscal tears (31% vs. 24%, *p* = 0.026). Independent risk factors for graft rupture included PTS ≥ 12° (odds ratio [OR] 3.1; 95% confidence interval [CI], 1.6–6.3; *p* < 0.001) and SATT ≥ 5 mm (OR 2.6; 95% CI, 1.2–5.5; *p* = 0.027), whereas neither ΔDATT nor absolute DATT was significantly associated with graft rupture.

**Conclusion:**

Preoperative DATT is not predictive of graft rupture following ACLR using hamstring autograft. PTS and SATT remain stronger predictors and should be prioritised for preoperative risk stratification.

**Level of Evidence:**

Level III, retrospective case‐control study.

## Graphical abstract

Graphical abstract showing that dynamic anterior tibial translation (DATT) assessed by instrumented laximetry appears to be more useful as a diagnostic tool than as a prognostic marker of anterior cruciate ligament graft rupture, unlike posterior tibial slope (PTS) and static anterior tibial translation (SATT), which were more strongly associated with graft rupture risk.

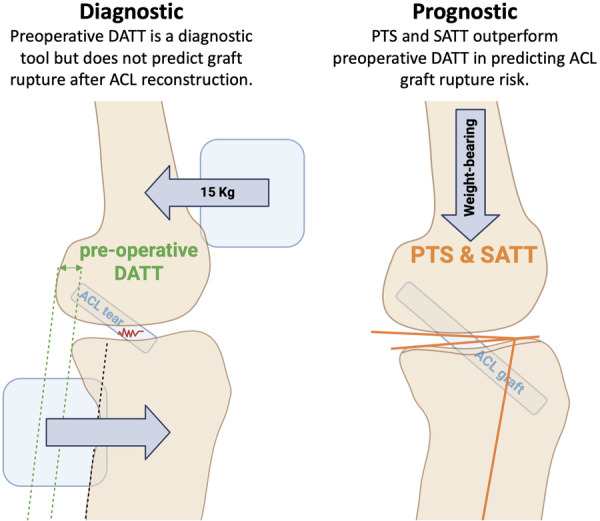

**Abbreviations**
ACL: anterior cruciate ligament
ACLR: anterior cruciate ligament reconstruction
ATT: anterior tibial translation
DATT: dynamic anterior tibial translation
LET: lateral extra‐articular tenodesis
LM: lateral meniscus
MM: medial meniscus
MRI: magnetic resonance imaging
OR: odds ratios
PTS: posterior tibial slope
ROM: range‐of‐motion
SATT: static anterior tibial translation
ΔDATT: side to side difference DATT

## INTRODUCTION

The anteroposterior laxity in the setting of anterior cruciate ligament (ACL) is typically assessed clinically with the Lachman test and quantified objectively through instrumented measurements of anterior tibial translation (ATT) [27]. Static ATT (SATT) can be assessed on lateral monopodal weight‐bearing radiographs as part of the evaluation of posterior tibial slope (PTS) and overall sagittal knee laxity [6, 34]. Dynamic ATT (DATT) is measured using instrumented devices such as the KT‐1000, Rolimeter™, GNRB, or Telos™, which quantify constraint anterior knee laxity under standardised loading conditions [4, 6, 21, 31]. Rotational laxity is commonly evaluated with the pivot‐shift test, although objective quantification is possible with tools such as accelerometers or rotameters [23, 26].

DATT represents an objective radiographic quantification of the Lachman test, which is useful for diagnosing ACL injury, and increased values are typically reduced following ACL reconstruction (ACLR) [4, 34]. DATT measured with Telos is consistently lower in ACL‐intact knees compared with ACL‐deficient knees [3]. Although highly specific but less sensitive for ACL tears, laximetry has demonstrated excellent intra‐ and interobserver reliability [3, 25]. Preoperative laximetry can also help characterise different patterns of ACL injury. Dejour et al. reported that a ΔDATT < 4 mm indicates a partial ACL tear with functional fibres, values between 4 and 9 mm reflect partial tears with nonfunctional fibres, and values > 9 mm correspond to complete ACL tears [10]. DATT has therefore demonstrated diagnostic validity. However, whether this objective quantification of anterior laxity translates into prognostic information regarding long‐term graft survival remains unclear.

Postoperative laximetry has also provided relevant insights. Increased anteroposterior laxity has been observed after ACLR combined with medial meniscectomy [9]. Moreover, a post‐operative ΔDATT greater than 5 mm at 1 year post‐ACLR has been associated with a five‐fold higher risk of graft rupture as well as poorer quality‐of‐life outcomes and lower return‐to‐sport rates [15].

PTS and SATT have been identified as strong predictors of ACL graft failure [28]. Furthermore, Dejour et al. demonstrated that DATT increases with greater PTS, concomitant medial meniscal tears, and complete ACL tears, and decreases with older age [11]. However, the role of preoperative laximetry in predicting graft rupture after ACLR remains controversial. Distinguishing between the diagnostic validity of laximetry and its potential prognostic relevance is essential when evaluating its role in preoperative risk assessment.

The aim of this study was to determine whether increased preoperative DATT, assessed as both absolute values and side‐to‐side difference (ΔDATT), is associated with a higher risk of graft rupture following ACLR using hamstring autografts. It was hypothesised that higher preoperative DATT values would be associated with increased rates of graft rupture.

## METHODS

### Study design

This was a retrospective analysis of a consecutive series of ACLRs performed at a dedicated sports knee referral centre between January 2014 and December 2017. Inclusion criteria were primary single‐bundle ACLR with hamstring tendon autograft, age ≥14 years, and a minimum follow‐up of 6 years. The lower age limit reflects the organisation of care in the institution, as patients younger than 14 years are managed in a dedicated paediatric institution. To avoid graft‐related bias, only patients who underwent hamstring graft ACLR were included, as the use of other graft types was less frequent. During the study period, only 12 bone‐patellar tendon‐bone autografts were performed, precluding meaningful analysis of this subgroup. Exclusion criteria were revision ACLR, concomitant procedures such as osteotomy or cartilage surgery, multiligament knee injuries, neurological or rheumatological disorders, a history of contralateral ACL injury, and inadequate lateral radiographs without overlapping of the two condyles.

### Surgical technique

All patients underwent ACLR using a hamstring tendon autograft. The semitendinosus and gracilis tendons were harvested using an open stripper. The graft was configured in a 4‐ to 6‐strand construct, depending on tendon length, to achieve a uniform graft diameter between 8 and 9 mm. Femoral and tibial fixation were achieved using bioabsorbable interference screws (Ligafix; Sciences & BioMaterials [SBM]). The femoral tunnel was positioned at the native ACL footprint using an outside‐in technique to replicate the anatomic centre of both bundles within a single‐bundle reconstruction. Concomitant meniscal lesions were repaired whenever possible using all‐inside devices (Fast‐Fix; Smith & Nephew). Partial meniscectomy was performed when repair was not feasible, according to intraoperative assessment by the senior surgeon (D.H.D.).

### Lateral extra‐articular tenodesis (LET)

A selective approach for LET. It was performed systematically in patients younger than 18 years. This age threshold reflects an institutional protocol, as younger patients are consistently reported to have higher rates of graft rupture and reinjury [5, 14]. In adults, LET was selectively performed in cases of genu recurvatum >10°, generalised hyperlaxity (Beighton score ≥ 4/9), or pivot‐shift grade 2 or 3 [19]. The modified Lemaire technique was used, harvesting a 1 × 9 cm strip of the posterior portion of the iliotibial band, passing it deep to the lateral collateral ligament, and fixing it with a 7‐mm interference screw (Ligafix; SBM) in a femoral tunnel positioned 5 mm proximal and 5 mm posterior to the lateral collateral ligament insertion at 80° of knee flexion with the limb in neutral rotation [8, 12].

### Postoperative management

All patients were discharged on the day of surgery and followed the same rehabilitation protocol. Isometric quadriceps exercises and passive and active range‐of‐motion (ROM) exercises from 0° to 90° were initiated on the first postoperative day, with progressive advancement to full ROM by 6 weeks. No brace was used. Weight‐bearing as tolerated with crutches was permitted. Patients who underwent meniscal repair for radial or root tears followed a non‐weight‐bearing protocol, with restriction of knee flexion beyond 90° during the first 3 weeks. Patients with other types of meniscal tears were allowed weight‐bearing as tolerated with crutches. Return to sports was typically permitted at 9 months based on functional and isokinetic test results.

### Data collection

The following variables were recorded: patient age at the time of surgery, sex, laterality and whether a concomitant LET was performed. The type of meniscal tear identified during arthroscopy was recorded and categorised as medial meniscus (MM), lateral meniscus (LM), or combined medial and lateral meniscus (MM and LM) tears. In cases of ACL graft rupture, the interval between the index surgery and graft rupture was documented.

### Imaging assessment

All patients underwent standardised preoperative radiographic evaluation at the same institution.

**Figure 1:**
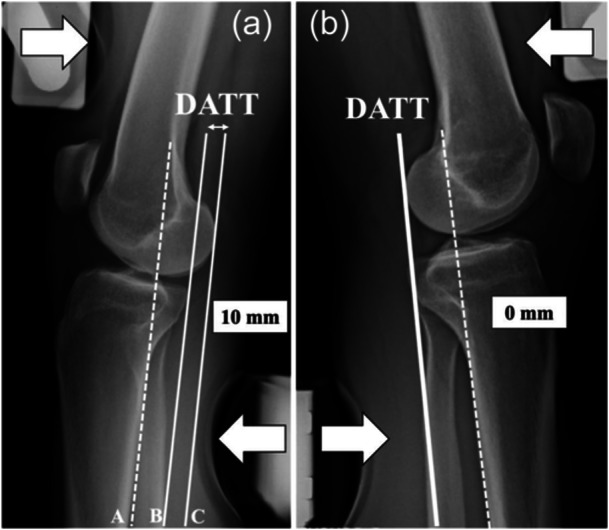
Telos™ stress lateral radiograph of the right anterior cruciate ligament (ACL)‐deficient knee (a) and the left uninjured knee (b) of the same patient. Two lines are traced parallel to the posterior tibial cortex (A). The first one is tangent to posterior part of the medial plateau (B) and the second one tangent to the medial femoral condyle (C). Dynamic anterior tibial translation (DATT) is the distance between these two lines (10 mm on the right knee and 0 mm on the left knee). ΔDATT is the side‐to‐side difference (10 mm). The two arrows represent the force (15 kg or 143 Newtons) applied to induce an anterior tibial translation.

DATT was measured using the Telos™ system with an anterior translation force of 15 kg or 147 Newtons (Figure 1) [3, 11]. DATT was defined as the distance between two lines drawn parallel to the posterior tibial cortex on stress: one tangent to the posterior aspect of the medial tibial plateau and the other tangent to the posterior contour of the medial femoral condyle under dynamic loading conditions. The side‐to‐side difference (ΔDATT) was calculated, with positive values indicating greater translation on the ACL‐deficient side. The absolute DATT value of the injured knee was also recorded. A positive value was recorded when the medial tibial plateau reference line was located anterior to the femoral condyle reference line, indicating ATT. Conversely, when the tibial plateau reference line was posterior to the femoral condyle reference line, the measurement was recorded as a negative value.

For PTS and SATT measurements, imaging included anteroposterior and true lateral monopodal weight‐bearing radiographs at 20° of knee flexion, with at least 15 cm of the proximal tibia visible. Measurements were performed on preoperative radiographs using HOROs DICOM viewer software (version 3.3.6). PTS was determined with the proximal anatomical axis method, defined as the angle between the perpendicular to the tibial diaphysis and the tangent to the anterior and posterior borders of the medial tibial plateau [28]. SATT was defined as the distance between two lines drawn parallel to the posterior tibial cortex, one tangent to the posterior margin of the medial tibial plateau and the other tangent to the posterior femoral condyles [6].

### Patient follow‐up

At a minimum follow‐up of 6 years, patients were contacted by phone and email to determine whether an ACL graft rupture had occurred after the index ACLR. Final follow‐up was defined as the time interval between ACL reconstruction and the last successful contact. ACL graft rupture was defined as clinical knee instability, characterised by a positive Lachman test with a soft endpoint and a positive pivot‐shift test, and graft rupture on magnetic resonance imaging (MRI) or arthroscopy. If graft rupture was managed at another institution, corresponding imaging reports (MRI) and/or operative reports were obtained to confirm the diagnosis. Patients were considered lost to follow‐up if no response was obtained after five phone calls and three emails over a 6‐month period. The follow‐up duration reported represents the maximum available in the database. Although most graft ruptures occur within the first two postoperative years, late ruptures can occur more than 10 years after surgery; therefore, the longest possible follow‐up was used [33].

### Subgroup analysis

Patients were stratified according to a ΔDATT threshold. Reported cutoffs in the literature vary from 2 to 6 mm [13, 25] because different methodology are used and no universal definition exists. In the present study, a cutoff of 6 mm was chosen, corresponding to the cohort's median ΔDATT value given its non‐normal distribution. Comparisons were performed between patients with ΔDATT <6 mm and ≥6 mm, as well as between those with and without ACL graft rupture. Additional analyses were conducted according to age group (<18 years vs. adults).

### Statistical analysis

Continuous variables are presented as mean ± standard deviation if normally distributed and as median ± interquartile range if non‐normally distributed. Categorical variables are expressed as counts and percentages. The Shapiro–Wilk test was applied to assess normality. Between‐group comparisons were performed using the chi‐square test or Fisher's exact test for categorical variables, and independent‐samples *t*‐test or Mann–Whitney *U* test for continuous variables, depending on the distribution. Spearman's rank correlation coefficients (*ρ*) were calculated to assess the relationships between absolute DATT and PTS, absolute DATT and SATT, ΔDATT and PTS, and ΔDATT and SATT. Risk factors for ACL graft rupture were first evaluated with univariate analyses, and then, in multivariable logistic regression. Odds ratios (ORs) with 95% confidence intervals (CIs) were reported. Fifty radiographs were independently reviewed to calculate the intraclass correlation coefficient (ICC) by two examiners (orthopaedic surgeons D.M. and N.C.). Intraobserver reliability was evaluated by repeating measurements twice by one examiner (D.M.) with a 2‐week interval. The ICC was calculated for PTS, SATT and DATT measurements. Given the number of graft rupture events observed in the cohort, the number of variables included in the multivariable logistic regression model was intentionally restricted to reduce the risk of overfitting. ΔDATT and DATT were also analysed as continuous variables in logistic regression models. As this was a retrospective study, all eligible patients available during the study period were included [2, 18]. All analyses were performed using SPSS Statistics (version 29.0.1.0; IBM Corp.). Statistical significance was set at *p* < 0.05.

## RESULTS

### Population

**Figure 2:**
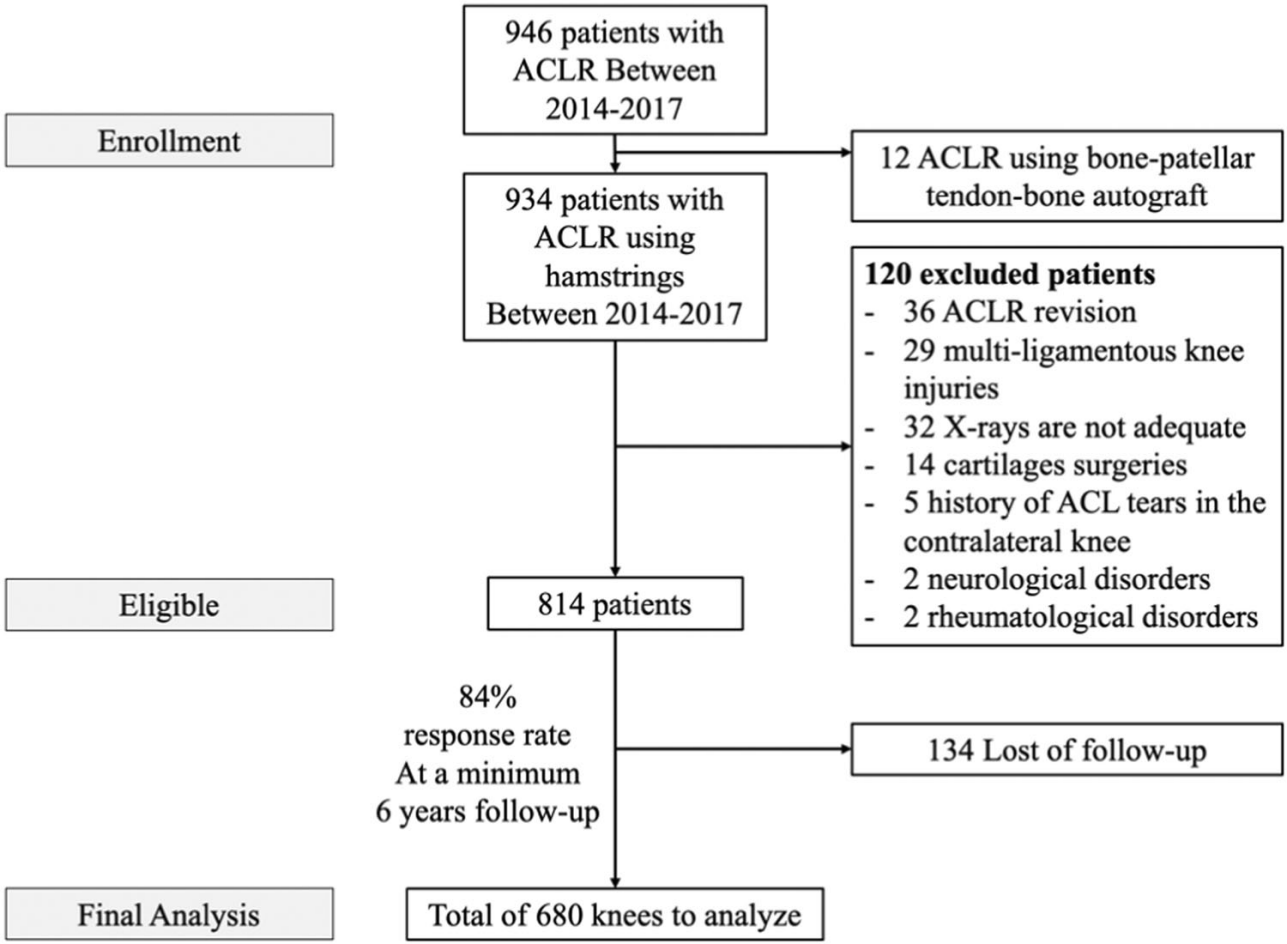
Flowchart of patients. ACL, anterior cruciate ligament; ACLR, anterior cruciate ligament reconstruction.

A total of 680 patients who underwent primary ACLR with hamstring autograft were included, with a mean follow‐up of 8.2 ± 1.7 years (range, 6–10 years). The study cohort is illustrated in the flowchart (Figure 2). Demographic characteristics and preoperative radiographic measurements of the overall cohort are summarised in Table 1. Forty‐one ACL graft ruptures (6%) were documented, occurring at a mean of 45 ± 30 months postoperatively (range, 7–115 months).

**Table 1:** Patients' demographics, ACL graft rupture, meniscus status and radiographic measurements.

Demographics	Mean ± SD (*n* = 680)	[Min–max]
Age (years)	30.5 ± 10.9	[14–71]
Gender (female/male)	261/419	
Side (right/left)	357/323	
LET	186 (27%)	
ACL graft rupture	41 (6%)	
MM tear	188 (28%)	
LM tear	105 (15%)	
MM and LM tears	87 (13%)	
Radiographic measurements		
PTS (°)	9.3 ± 2.4	[2–18]
SATT (mm)	2.4 ± 3.5	[–2 to 14]
DATT (mm)^a^	9 ± 5	[0–23]
ΔDATT (mm)^a^	6 ± 4	[0–16]

Abbreviations: ACL, anterior cruciate ligament; DATT, dynamic anterior tibial translation; LET, lateral extra‐articular tenodesis; LM, lateral meniscus; MM, medial meniscus; PTS, posterior tibial slope; SATT, static anterior tibial translation; ΔDATT, dynamic anterior tibial translation side‐to‐side difference.

^a^ Present as median and interquartile range.

### Subgroups analysis

Table 2 presents the comparison of the cohort regarding the threshold of 6 mm. Patients with ΔDATT ≥ 6 mm presented a higher prevalence of medial meniscal tears intraoperatively.

**Table 2:** Comparison between patients using ΔDATT threshold of 6 mm.

	ΔDATT < 6 mm (48%, *n* = 329)	ΔDATT ≥ 6 mm (52%, *n* = 351)	*p*‐Value
Age (years)	32 ± 11	29 ± 11	**<0.001**
Gender (female, %)	134 (41%)	127 (36%)	0.223
LET	58 (18%)	128 (36%)	**<0.001**
PTS	9 ± 2.4	9.5 ± 2.5	**0.011**
SATT	2 ± 3.5	2.8 ± 3.6	**0.005**
ACL graft rupture rate	19 (5.8%)	22 (6.3%)	0.787
MM tear	78 (24%)	110 (31%)	**0.026**
LM tear	52 (16%)	53 (15%)	0.799
MM and LM tear	32 (10%)	55 (16%)	**0.020**

*Note*: Statistically significant variables are presented in bold (*p* < 0.05).

Abbreviations: ACL, anterior cruciate ligament; LET, lateral extra‐articular tenodesis; LM, lateral meniscus; MM, medial meniscus; PTS, posterior tibial slope; SATT, static anterior tibial translation; ΔDATT, dynamic anterior tibial translation side‐to‐side difference.

Comparison between the 41 patients with a graft rupture and the rest of the cohort is present in Table 3.

**Table 3:** Comparison between patients with and without an ACL graft rupture.

	ACL graft rupture (*n* = 41)	No ACL graft rupture (*n* = 639)	*p*‐Value
Age (years)	23 ± 8	31 ± 11	**<0.001**
Gender (female %)	39%	38%	0.931
LET	27%	27%	0.957
PTS (mean ± SD)	10.8 ± 2.2	**9.2** ± **2.4**	**<0.001**
SATT (mean ± SD)	4.3 ± 3.7	**2.3** ± **3.5**	**<0.001**
DATT (median, IQR)	10 [5]	9 [5]	0.272
ΔDATT (median, IQR)	6 [4]	6 [4]	0.211

*Note*: Statistically significant variables are presented in bold (*p* < 0.05).

Abbreviations: ACL, anterior cruciate ligament; DATT, dynamic anterior tibial translation; IQR, interquartile range; LET, lateral extra‐articular tenodesis; PTS, posterior tibial slope; SATT, static anterior tibial translation; ΔDATT, dynamic anterior tibial translation side‐to‐side difference.

Subgroup comparisons between patients <18 years and adults are summarised in Table 4.

**Table 4:** Age comparison.

	<18 years old (n = 69)	≥18 years old (*n* = 611)	*p*‐Value
Gender (female %)	42 (61%)	219 (36%)	**<0.001**
ACL graft rupture	8 (11.6%)	33 (5.4%)	**0.040**
PTS (mean ± SD)	9.5 ± 2.3	9.3 ± 2.5	0.380
SATT (mean ± SD)	2 ± 3.5	2.5 ± 3.5	0.334
DATT (median ± IQR)	12 [4]	9 [5]	**<0.001**
ΔDATT (median ± IQR)	8 [5]	5 [4]	**<0.001**

*Note*: Statistically significant variables are presented in bold (*p* < 0.05).

Abbreviations: ACL, anterior cruciate ligament; DATT, dynamic anterior tibial translation; IQR, interquartile range; PTS, posterior tibial slope; SATT, static anterior tibial translation; ΔDATT, dynamic anterior tibial translation side‐to‐side difference.

### Correlation between radiographic measurements

**Figure 3:**
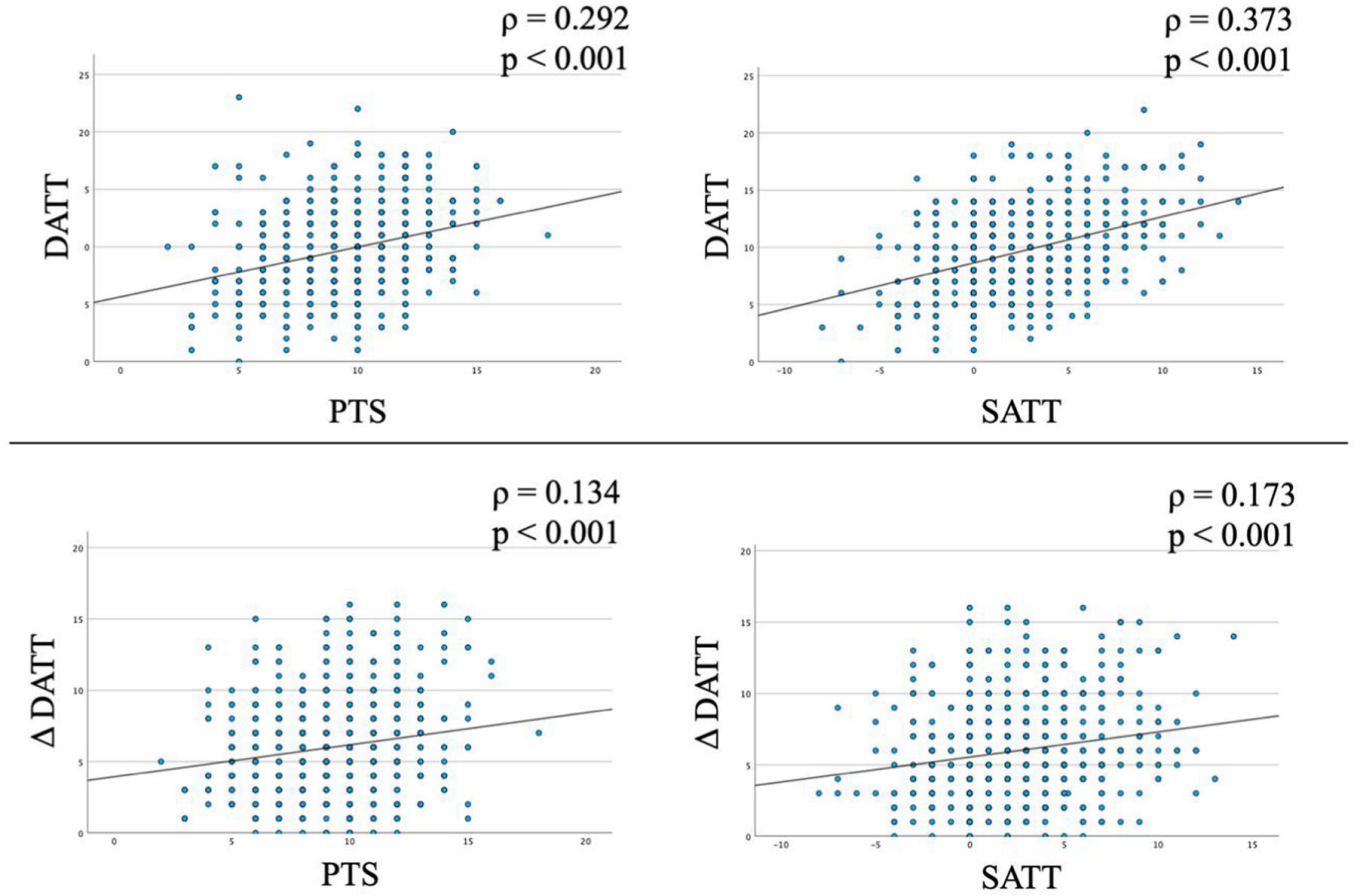
Scatter plots of absolute DATT and ΔDATT values according to PTS and SATT. DATT, dynamic anterior tibial translation; ΔDATT, side‐to‐side difference in dynamic anterior tibial translation; PTS, posterior tibial slope; SATT, static anterior tibial translation; *ρ*, Spearman's rank correlation coefficient.

DATT, both in absolute value and side‐to‐side difference, demonstrated significant correlations with PTS and SATT (Figure 3). These correlations were stronger for absolute DATT values.

### Risk factors analysis

The results of the multivariable logistic regression analysis are presented in Table 5. ΔDATT ≥ 6 mm (*p* = 0.643), female sex (*p* = 0.882) and LET (*p* = 0.384) were not independently associated with ACL graft rupture.

**Table 5:** Multivariate logistic analysis of risk factors for anterior cruciate ligament graft rupture.

Variable	OR	95% CI	*p*‐Value
PTS ≥ 12°	3.1	1.6–6.3	**<0.001**
SATT ≥ 5 mm	2.6	1.2–5.5	**0.027**
Age < 18	2.3	1.1–3.9	**0.036**
LM tear	2.4	1–4.9	**0.040**

*Note*: Statistically significant variables are presented in bold (*p* < 0.05). Abbreviations: DATT, dynamic anterior tibial translation; OR, odds ratio; PTS, posterior tibial slope; SATT, static anterior tibial translation.

When analysed as continuous variables, neither ΔDATT (OR, 1.05; 95% CI, 0.96–1.13; *p* = 0.256) nor absolute DATT (OR, 1.04; 95% CI, 0.96–1.13; *p* = 0.260) was independently associated with ACL graft rupture.

There were excellent intra‐ and interobserver reliability ICCs for PTS (0.930 and 0.922, respectively), SATT (0.965 and 0.920, respectively) and DATT (0.918 and 0.908, respectively) measurements.

## DISCUSSION

The principal finding of this study is that neither preoperative DATT nor ΔDATT independently predicted ACL graft rupture at a minimum 6‐year follow‐up. This absence of association persisted when DATT and ΔDATT were analysed as continuous variables, suggesting that the lack of prognostic value was not related to dichotomisation or the 6 mm threshold selection. Higher values of DATT were associated with increased PTS and SATT, two other parameters already recognised as major risk factors for graft rupture [28]. Several confounding factors may explain this observation. Dejour et al. demonstrate that pre‐operative DATT increases with greater PTS, concomitant medial meniscal tears, and complete ACL ruptures, while decreasing with age [11]. Given its dependence on multiple variables, DATT appears unreliable for predicting the risk of ACL graft rupture, whereas PTS and SATT remain independent and consistent predictors [28, 33]. Although not predictive of ACL graft rupture, pre‐operative laximetry remains clinically useful. It can assist surgeons in identifying the presence of residual functional fibres in partial ACL tears and help guide the indication for surgery [10]. Previous studies using Telos™ stress radiography and other instrumented devices have primarily focused on the diagnostic performance of anterior laxity assessment rather than its prognostic implications [24, 32]. Also, Klasan et al. reported strong reliability and reproducibility of instrumented anterior translation measurements [22]. These findings are consistent with the present results, suggesting that instrumented laximetry remains a robust diagnostic tool but should not be considered a standalone predictor of graft rupture risk.

No association towards a higher graft rupture rate was observed in patients with ΔDATT ≥ 6 mm. However, this subgroup also had a significantly higher rate of LET, which is known to reduce graft rupture risk and may have masked the association [7, 16]. ΔDATT itself was not a direct factor in the indication for LET. In this selective ‘à la carte’ approach, the decision to perform LET is primarily based on age, pivot‐shift grade, and generalised hyperlaxity, factors that also influence DATT. This likely explains the higher prevalence of LET in patients with increased ΔDATT [10, 11]. As shown in previous study, the ‘à la carte’ approach highlights that LET is not a universal solution capable of eliminating ACL graft rupture, but rather a selective adjunct to reduce risk in chosen patients [28].

In the ACL‐deficient knee, the MM becomes the primary stabiliser of anteroposterior translation [1]. This may explain why patients with higher ΔDATT values were more likely to present MM tears, although the precise tear pattern was not documented. Ramp lesions, in particular, have been associated with increased anterior translation [35]. LM is more closely related to rotational knee stability, which likely explains why laximetry based solely on ATT does not demonstrate an association LM tear [29]. DATT measures the translation of the medial tibial plateau relative to the medial femoral condyle, which may explain its closer link with the medial compartment [11]. In the present study, LM tear was also associated with ACL graft rupture (OR 2.5; 95% CI, 1–5.8), consistent with previous reports highlighting the detrimental effect of LM injury on rotational stability [17, 28].

Fiil et al. reported that a post‐operative ΔDATT > 5 mm 1 year postoperatively was a significant risk factor for ACL graft rupture at 2 years, with an almost 18% rupture rate in this subgroup [15]. In the same study, younger patients demonstrated higher DATT values. In the present cohort, younger patients demonstrated higher preoperative absolute and ΔDATT values, along with higher ACL graft rupture rates. Age is a well‐known risk factor for graft rupture, and its association with DATT may be explained by greater knee anterior laxity in younger patients [20, 30, 36].

Regarding correlations between radiographic parameters, absolute DATT was more strongly correlated with PTS and SATT than ΔDATT. This is expected, as absolute values are measured only on the injured side, consistent with PTS and SATT. ΔDATT reflects differences with the contralateral knee, which is not analysed by PTS or SATT [6].

Some limitations of this study should be acknowledged. It was retrospective study with the inherent biases of this design. Sixteen per cent of patients were lost to follow‐up, which may be explained by difficulties in maintaining long‐term contact with younger patients. However, the maximum number of available patients was used in this retrospective analysis. Although the overall cohort was large, the number of graft rupture events was limited, which may affect model stability despite efforts to restrict covariate inclusion. Additionally, the higher prevalence of LET in the ΔDATT ≥ 6 mm subgroup may have influenced graft rupture rates and potentially masked an association between preoperative dynamic laxity and graft failure. Only hamstring autografts were studied, which limits the generalisability of the findings to other graft types. No data were available on the precise type of meniscal tears, patient activity level, clinical outcomes, tunnel position, or time from injury to surgery, all of which may influence the risk of graft rupture. No postoperative DATT measurements were available, and future studies should investigate the correlation between these values and graft rupture rate.

## CONCLUSION

Preoperative DATT is not predictive of graft rupture following ACLR using hamstrings and with a selective approach for LET. Telos‐based instrumented laximetry remains a diagnostic tool for assessing ACL injury severity but should not be considered a prognostic indicator of graft survival. Other quantifiable preoperative parameters, such as PTS and SATT, provide stronger prognostic information.

## AUTHOR CONTRIBUTIONS

David Mazy, Nicolas Cance, Lucia Angelelli, Tomas Pineda and Michael James Dan drafted the manuscript. David Mazy and David Henri Dejour were responsible for the research design. David Mazy, Nicolas Cance, Lucia Angelelli, Tomas Pineda and Michael James Dan were responsible for data acquisition. David Mazy, Nicolas Cance, Lucia Angelelli, Tomas Pineda, Michael James Dan and David Henri Dejour analysed and interpreted the data. All authors reviewed and approved the final manuscript.

## CONFLICT OF INTEREST STATEMENT

David Henri Dejour has received royalties from Arthrex, Science & BioMaterials (SBM), and Corin; and consulting fees from Smith & Nephew. The remaining authors declare no conflicts of interest.

## ETHICS STATEMENT

All patients were prospectively enrolled in an institutional registry and provided informed consent at the time of inclusion for the use of their data for research purposes. The study protocol was approved by the institutional review board (No. COS‐RGDS‐2020‐03‐006‐DEJOUR‐D).

## Data Availability

The data that support the findings of this study are available on request from the corresponding author. The data are not publicly available due to privacy or ethical restrictions.
